# Skin Cancer Predisposition Genes, Full‐Body Skin Examinations, Familial Disclosure, and Genetic Testing Among High‐Risk Individuals

**DOI:** 10.1111/ijd.70206

**Published:** 2025-12-10

**Authors:** Jincong Q. Freeman, Yu Matsui, Fangyuan Zhao, Olufunmilayo I. Olopade, Dezheng Huo

**Affiliations:** ^1^ Department of Public Health Sciences The University of Chicago Chicago Illinois USA; ^2^ Cancer Prevention and Control Research Program UChicago Medicine Comprehensive Cancer Center Chicago Illinois USA; ^3^ Center for Health and the Social Sciences The University of Chicago Chicago Illinois USA; ^4^ Department of Dermatology, Faculty of Medicine Academic Assembly, University of Toyama Toyama Japan; ^5^ Center for Innovation in Global Health The University of Chicago Chicago Illinois USA; ^6^ Key Laboratory of Carcinogenesis and Translational Research (Ministry of Education/Beijing), Laboratory of Molecular Oncology Peking University Cancer Hospital & Institute Beijing China; ^7^ Section of Hematology/Oncology, Department of Medicine The University of Chicago Chicago Illinois USA

**Keywords:** familial disclosure, full‐body skin examination, genetic testing, high‐risk populations, PV/LPV, skin cancer predisposition genes

## Abstract

**Background:**

There is a lack of knowledge in full‐body skin examinations (FBSEs) in the context of pathogenic or likely pathogenic variants (PV/LPV) in skin cancer predisposition genes (CPGs). This study assessed the association between carrier status of PV/LPV in skin CPGs and FBSEs and described the patterns of family letter receipt, familial disclosure, and cascade testing among high‐risk individuals.

**Methods:**

Between July and September 2023, we surveyed participants enrolled in the Chicago Cancer Prone Study. Carrier status of PV/LPV in skin CPGs was defined as having been told by a genetic counselor that they have a PV/LPV in *CDKN2A*, *TP53*, *PTEN*, *BRIP1*, *PALB2*, *ATM*, and/or *CHEK2* genes. FBSEs were assessed by asking individuals whether they ever had an FBSE and modeled using logistic regression.

**Results:**

Of 579 individuals (mean age, 59.5 years), 11.6% carry PV/LPV in skin CPGs and 63.8% ever had a FBSE. Carriers had greater odds of FBSEs than non‐carriers (adjusted odds ratio, 2.15; 95% CI, 1.03–4.52). White race, older age, and female sex were associated with increased odds of FBSEs. Among carriers, 65.3% received a family letter from their genetic counselor after testing; 91.0% disclosed their PV/LPV‐carrying status to any family members, and 62.7% of the individuals' family members underwent genetic testing.

**Conclusions:**

We found greater odds of FBSEs among PV/LPV carriers, notable disparities in FBSEs, and prevalent family letter receipt, familial disclosure, and cascade testing. This study highlights the role of PV/LPV‐carrying status on FBSEs and the potential need for comprehensive guidelines beyond known risk factors to optimize skin cancer screening in high‐risk populations.

## 1 Introduction

Skin cancer is the most common type of cancer in the United States (US) [1]. In 2024, an estimated 100,640 new cases of invasive malignant melanoma (MM) and 99,700 cases of melanoma in situ were reported in the US, with 8290 MM‐related deaths attributed to invasive melanoma [2]. For nonmelanoma skin cancer (NMSC), including squamous cell carcinoma (SCC) and basal cell carcinoma (BCC), the overall incidence was estimated to reach approximately 5.4 million adult cases, which was derived based on 2012 US population data [3]. An analysis of the 2012–2018 Medical Expenditure Panel Survey data reported that approximately 6 million adults underwent skin cancer treatment, associating with an estimated total cost of $8.9 billion in the US each year [4]. Understanding disease risk and providing appropriate follow‐up care are crucial for preventing metastasis. This approach is key to facilitating timely surgical excision and identifying patients eligible for systemic adjuvant therapies, ultimately improving health outcomes. The primary rationale for screening is to detect skin cancer early, enabling timely and effective treatment to reduce skin cancer‐related mortality. One simple yet effective method is population‐based skin cancer screening by dermatologists, such as full‐body skin examinations (FBSEs).

An annual FBSE is not currently recommended as a screening or follow‐up measure by major professional organizations. The American Academy of Dermatology (AAD) recommends that high‐risk patients, particularly those with a history of NMSC, should undergo annual screenings for new keratinocyte cancers and melanoma. They also emphasize that high‐risk patients should be counseled on the elevated risk of developing new primary skin cancer, the importance of regular in‐office screenings by a dermatologist, and the potential benefits of self‐screening [5, 6, 7]. In contrast, the US Preventive Services Task Force conducted a panel review and concluded: “the evidence is insufficient, and the balance of benefits and harms for visual skin examination by a clinician to screen for skin cancer in asymptomatic adolescents and adults cannot be determined” [8]. This reflects ongoing debates that concern its utility in secondary prevention. A significant gap exists between asymptomatic individuals and those at high risk for skin cancer. Many skin cancers result from a complex interplay between environmental factors and individual genetic susceptibility. Risk factors for MM include multiple melanocytic nevi, fair skin, intermittent sun exposure, and a family history of melanoma [9, 10, 11]. Most melanomas are sporadic, accounting for approximately 90% of cases and occurring in individuals without a family history [12], and an estimated 1%–10% are those individuals with highly penetrant susceptibility genes [13, 14]. Although small in proportion, this group exists as a clinically significant population.

There were several high‐penetrance and low‐to‐moderate penetrance genes for skin cancers. Briefly, *CDKN2A*, a high‐risk gene for familial MM encoding *p16INK4A* and *p14ARF*, regulates CDK4/6 and *p53*. Pathogenic variant or likely pathogenic variant (PV/LPV) disrupts these tumor suppressor pathways, promoting MM development and potentially contributing to early‐stage SCC [15, 16, 17, 18]. *TP53* is a tumor suppressor encoding p53 and regulates apoptosis, DNA repair, and the cell cycle, and germline PV/LPVs in *TP53* cause Li‐Fraumeni syndrome and increase susceptibility to MM and SCC [19, 20, 21, 22]. PV/LPVs in *PTEN*, a tumor suppressor regulating cell survival, cause Cowden syndrome and slightly increase MM risk, though their role in MM remains less emphasized compared to *CDKN2A* and *TP53* [23, 24, 25]. *BRIP1* is a DNA helicase involved in *BRCA1*‐mediated repair. Though mainly linked to ovarian cancer, rare PV/LPVs in *BRIP1* have been observed in melanocytic tumors, suggesting a possible hereditary MM risk [26, 27, 28, 29]. *PALB2* is a key *BRCA1*/*2* partner facilitating homologous recombination. While its role in MM remains uncertain, PV/LPVs in *PALB2* have been associated with SCC and other NMSC [30, 31, 32, 33]. *ATM* is a DNA damage repair gene whose germline PV/LPVs moderately increase MM risk, especially in high‐risk individuals. Rarely, these variants have also been linked to early‐onset Merkel cell carcinoma [34, 35, 36]. PV/LPVs in *CHEK2*, a cell cycle checkpoint kinase involved in the DNA damage response, have been linked to a moderate MM risk and an increased susceptibility to BCC, likely due to impaired DNA repair [20, 37, 38]. *BRCA1*/*2* genes are well‐documented susceptibility genes for breast and ovarian cancers, but their roles in skin cancer remain controversial. While some studies have linked them to an increased risk of skin cancer and melanoma [39, 40], others found insufficient evidence to support this assocation [41, 42].

As genetic research continues to identify new cancer predisposition genes (CPGs), defining the appropriate population for skin cancer screening becomes increasingly important. In particular, individuals carrying a PV/LPV, even if asymptomatic, may benefit from routine FBSEs. However, there is currently no consensus on how to define “asymptomatic” individuals eligible for screening, which creates ambiguity in clinical practice. Despite growing awareness, there is a lack of knowledge regarding the role of FBSEs in the context of PV/LPV in skin CPGs in high‐risk populations. To address the existing literature gap, we examined (1) the association between carrier status of PV/LPV in skin CPGs and having an FBSE and (2) the patterns of family letter receipt, familial disclosure, and cascade testing in a multiethnic cohort of high‐risk individuals.

## 2 Materials and Methods

### 2.1 Design, Setting, and Participants

This was a cross‐sectional study and an exploratory analysis. Between July and September 2023, we conducted a survey among individuals enrolled in the Chicago Cancer Prone Study (CCPS). Briefly, CCPS is a local registry of diverse high‐risk families at a hospital‐based preventive oncology clinic that has registered 5685 participants as of September 2024 since its establishment in 1992. All study participants provided written informed consent to CCPS and follow‐up surveys. The University of Chicago Institutional Review Board reviewed and approved this study. We followed the Strengthening the Reporting of Observational Studies in Epidemiology (STROBE) reporting guideline [43].

### 2.2 Eligibility and Cohort Selection

Figure S1 depicts the sample selection of individuals aged ≥ 18 years from CCPS. Briefly, we sent 2404 email invitations and 611 mail invitations to participants with contact information documented in the registry between July and September 2023. A total of 912 participants responded via REDCap online or paper questionnaires. We further excluded individuals who reported carrying PV/LPV in non‐skin cancer susceptibility genes and obtained a final sample size of 579 for the main analysis. In our sensitivity analysis, we included *BRCA1*/*2* data: 69 *BRCA1* carriers, 59 *BRCA2* carriers, and 1 individual carrying PV/LPVs in both *BRCA1*/*2* genes.

### 2.3 Measures

The main outcome of interest was FBSEs, which was assessed by asking participants whether they ever had an FBSE. Among those who had FBSEs, we asked how long it has been since the most recent skin exam. CCPS registry participants underwent genetic testing and counseling upon enrollment, and the average number of years between genetic testing and the current study was 10 years. Participants may not be aware of their PV/LPV status and skin cancer risk before enrollment. Therefore, they likely received an FBSE after genetic testing and counseling. Individuals were also asked whether they had ever been told by a doctor or health care provider that they were at risk for skin cancer, including sun exposure, indoor tanning, prior history of skin cancer, a family history of skin cancer, and lots of abnormal moles or pigmented surface areas.

The main exposure of interest was carrier status of PV/LPV in skin CPGs, defined as having been told by a genetic counselor or clinician that they had a PV/LPV that increases the risk of developing cancer; and if yes, which deleterious PV/LPV they currently carry. Our clinical investigators verified self‐reported PV/LPVs and confirmed them through registry records. Carriers were also asked whether they had (1) received a family letter from their genetic counselor or other clinicians after genetic testing, (2) shared the letter with at least one family member, (3) disclosed the identified PV/LPV to their family members, and (4) whether any of their family members underwent genetic testing for the identified gene variant(s); and if yes, how or where they received the genetic testing. We investigated a range of PV/LPVs in skin CPGs identified in our cohort, including high‐penetrance genes (*CDKN2A*, *TP53*, and *PTEN*) and low‐to‐moderate penetrance genes (*BRIP1*, *PALB2*, *ATM*, and *CHEK2*). We included *BRCA1*/*2* data in the sensitivity analysis.

Demographic and socioeconomic characteristics included age at survey, sex assigned at birth, race/ethnicity (non‐Hispanic Asian, non‐Hispanic Black, non‐Hispanic White, or Hispanic), marital status, highest level of education (higher school/GED or less, some college or Associate's degree, Bachelor's degree, and graduate or professional degree), smoking status, type of health insurance (Medicaid, Medicare, private, other, or uninsured), and annual household income.

### 2.4 Statistical Analysis

Summary statistics were used to describe the study cohort, overall and by PV/LPV carrier status. Bivariate analyses were performed using *t*‐tests or Wilcoxon rank‐sum tests for continuous variables and Pearson's chi‐squared or Fisher's exact tests for categorical variables. Multivariable logistic regression was used to examine the difference in FBSEs by PV/LPV carrier status. We fit several models. Model 1 included carrier status of PV/LPV in skin CPGs and age at survey. In Model 2, we further controlled for sex, race/ethnicity, highest level of education, annual household income, and type of health insurance. In our sensitivity analysis, we included individuals carrying PV/LPVs in the *BRCA1* and/or *BRCA2* genes in the carrier category and utilized the same regression approach. Adjusted odds ratios (AOR) and 95% CIs were calculated. All statistical tests were 2‐sided, and *p* values < 0.05 were considered statistically significant. All analyses were performed using Stata 18.0 (StataCorp) from December 2023 to January 2025.

## 3 Results

### 3.1 Characteristics of the Study Sample

Of 579 high‐risk individuals, the mean (SD) age was 59.5 (13.1) years; 81.9% were female; 3.0% were Asian, 11.4% were Black, 4.1% were Hispanic, and 81.5% were White; 31.1% had a Bachelor's degree and 45.4% had a graduate or professional degree; 61.3% were privately insured while 33.0% were on Medicare. Overall, 67 (11.6%) carry PV/LPV in skin CPGs (Table 1). For high penetrance genes, 6, 4, and 1 individuals carry PV/LPVs in *CDKN2A*, *TP53*, and *PTEN*, respectively. For low‐to‐moderate penetrance genes, 24, 17, 12, and 2 individuals carry PV/LPVs in *CHEK2*, *ATM*, *PALB2*, and *BRIP1*, respectively (Figure S2).

**TABLE 1:** Sociodemographic characteristics of high‐risk individuals, overall and by carrier status of pathogenic/likely pathogenic variants in skin cancer predisposition genes.

Characteristics	Overall	Carrier status of PV/LPV in skin cancer predisposition genes		*p* ^a^
		Non‐carriers	Carriers	
	*N* = 579	*n* = 512 (88.4%)	*n* = 67 (11.6%)	
	No. (%)	No. (%)	No. (%)	
Age (in years)				
Mean (SD)	59.5 (13.1)	60.0 (13.0)	55.7 (13.2)	0.01
Median (IQR)	61.0 (50.0, 70.0)	61.0 (51.0, 70.0)	56.0 (45.0, 65.0)	0.01
Sex assigned at birth				
Female	474 (81.9)	420 (82.0)	54 (80.6)	0.77
Male	105 (18.1)	92 (18.0)	13 (19.4)	
Race/ethnicity				
Non‐Hispanic Asian	17 (3.0)	15 (3.0)	2 (3.1)	0.72
Non‐Hispanic Black	64 (11.4)	58 (11.7)	6 (9.2)	
Non‐Hispanic White	457 (81.5)	404 (81.5)	53 (81.5)	
Hispanic	23 (4.1)	19 (3.8)	4 (6.2)	
Marital status				
Married or living with a partner	404 (72.0)	352 (71.1)	52 (78.8)	0.46
Single or never married	55 (9.8)	50 (10.1)	5 (7.6)	
Widowed, divorced or separated	102 (18.2)	93 (18.8)	9 (13.6)	
Highest level of education				
High school, GED or less	42 (7.5)	35 (7.0)	7 (10.8)	0.69
Some college or Associate's degree	90 (16.0)	80 (16.1)	10 (15.4)	
Bachelor's degree	175 (31.1)	157 (31.6)	18 (27.7)	
Graduate or professional degree	255 (45.4)	225 (45.3)	30 (46.2)	
Type of health insurance				
Private	344 (61.3)	299 (60.3)	45 (69.2)	0.44
Medicaid	3 (0.5)	3 (0.6)	0	
Medicare	185 (33.0)	169 (34.1)	16 (24.6)	
Other or other government plan	24 (4.3)	20 (4.0)	4 (6.2)	
Uninsured	5 (0.9)	5 (1.0)	0	
Annual household income				
< $50,000	50 (9.5)	45 (9.7)	5 (8.1)	0.77
$50,000–$74,999	59 (11.2)	51 (11.0)	8 (12.9)	
$75,000–$99,999	66 (12.5)	55 (11.9)	11 (17.7)	
$100,000–$149,999	116 (22.1)	103 (22.2)	13 (21.0)	
$150,000–$199,999	73 (13.9)	64 (13.8)	9 (14.5)	
≥ $200,000	162 (30.8)	146 (31.5)	16 (25.8)	
Smoking status				
Current	14 (2.6)	13 (2.7)	1 (1.5)	0.86
Past	147 (27.1)	131 (27.4)	16 (24.6)	
Never	382 (70.3)	334 (69.9)	48 (73.8)	
Ever told by a doctor or other health care provider that you are at risk for skin cancer				
No	361 (67.6)	333 (70.5)	28 (45.2)	< 0.001
Yes	173 (32.4)	139 (29.5)	34 (54.8)	
Ever had a full‐body skin examination				
No	194 (36.2)	177 (37.5)	17 (26.6)	0.09
Yes	342 (63.8)	295 (62.5)	47 (73.4)	

Abbreviations: GED, general education development; IQR, interquartile range; No., number; PV/LPV, pathogenic/likely pathogenic variants; SD, standard deviation.

^a^ *p* values were calculated using Student's *t*, Wilcoxon rank‐sum, Pearson's chi‐squared, or Fisher's exact tests, as appropriate.

Compared to non‐carriers of PV/LPV in skin CPGs, carriers were younger (mean [SD] age, 55.7 [13.2] vs. 60.0 [13.0] years; *p* = 0.01). A greater proportion of PV/LPV carriers reported having been told by a doctor or other health provider that they were at risk for skin cancer than that of non‐carriers (54.8% vs. 29.5%; *p* < 0.001) (Table 1). Among carriers who were told they were at high risk, 40.3% reported sun exposure, followed by 29.9% family history, 20.9% personal history, 17.9% lots of abnormal moles or pigmented areas, and 7.5% indoor tanning. When including *BRCA1*/*2* data, similar distributions of sociodemographic characteristics were observed (Table S1).

### 3.2 Carrier Status and Full‐Body Skin Examinations

Overall, 63.8% of the high‐risk individuals reported ever having had an FBSE. A slightly higher percentage of PV/LPV carriers had an FBSE compared to non‐carriers (73.4% vs. 62.5%; *p* = 0.09) (Table 1). In the age‐adjusted model (Model 1), carriers had greater odds of FBSEs than non‐carriers (AOR, 1.88; 95% CI, 1.03–3.41; *p* = 0.04). When additionally controlled for sociodemographic characteristics (Model 2), carriers remained at greater odds of FBSEs than non‐carriers (AOR, 2.15; 95% CI, 1.03–4.52; *p* = 0.04) (Table 2). In the same model, age (AOR, 1.38; 95% CI, 1.10–1.74; *p* = 0.006) and female sex (AOR, 2.42; 95% CI, 1.38–4.24; *p* = 0.002) were significantly associated with increased odds of FBSEs. Asian (AOR, 0.18; 95% CI, 0.06–0.57; *p* = 0.003), Black (AOR, 0.03; 95% CI, 0.01–0.08; *p* < 0.001), or Hispanic (AOR, 0.12; 95% CI, 0.04–0.33; *p* < 0.001) individuals had significantly lower odds of FBSEs than White individuals. High school or less education (vs. graduate or professional degree) was associated with significantly lower odds of FBSEs (AOR, 0.19; 95% CI, 0.08–0.48; *p* < 0.001) (Table 2).

**TABLE 2:** Association between carrier status of pathogenic/likely pathogenic variants in skin cancer predisposition genes and full‐body skin examinations among high‐risk individuals.

Characteristics	Multivariable logistic regression			
	Model 1		Model 2	
	AOR (95% CI)^a^	*p*	AOR (95% CI)^b^	*p*
Carrier status of PV/LPV in skin cancer predisposition genes				
Carriers	1.88 (1.03–3.41)	0.04	2.15 (1.03–4.52)	0.04
Non‐carriers	1 [reference]		1 [reference]	
Age (in years)^c^	1.27 (1.11–1.46)	0.001	1.38 (1.10–1.74)	0.006
Sex assigned at birth				
Female	NA	NA	2.42 (1.38–4.24)	0.002
Male	NA	NA	1 [reference]	
Race/ethnicity				
Non‐Hispanic Asian	NA	NA	0.18 (0.06–0.57)	0.003
Non‐Hispanic Black	NA	NA	0.03 (0.01–0.08)	< 0.001
Non‐Hispanic White	NA	NA	1 [reference]	
Hispanic	NA	NA	0.12 (0.04–0.33)	< 0.001
Highest level of education				
High school, GED or less	NA	NA	0.19 (0.08–0.48)	< 0.001
Some college or Associate's degree	NA	NA	0.57 (0.29–1.11)	0.10
Bachelor's degree	NA	NA	0.71 (0.41–1.22)	0.22
Graduate or professional degree	NA	NA	1 [reference]	
Annual household income				
< $50,000	NA	NA	0.53 (0.20–1.37)	0.19
$50,000–$74,999	NA	NA	0.47 (0.20–1.11)	0.09
$75,000–$99,999	NA	NA	0.75 (0.34–1.65)	0.48
$100,000–$149,999	NA	NA	0.58 (0.31–1.10)	0.09
$150,000–$199,999	NA	NA	0.77 (0.37–1.59)	0.48
≥ $200,000	NA	NA	1 [reference]	
Type of health insurance				
Private	NA	NA	1 [reference]	
Medicaid/Medicare	NA	NA	1.01 (0.51–2.01)	0.98
Other or other government plan	NA	NA	0.67 (0.22–2.02)	0.48

Abbreviations: AOR, adjusted odds ratio; CI, confidence interval; GED, general education development; NA, not applicable; PV/LPV, pathogenic/likely pathogenic variants.

^a^ Adjusted for age only.

^b^ Adjusted for age, sex assigned at birth, race/ethnicity, highest level of education, annual household income, and type of health insurance coverage.

^c^ The AOR and 95% CI for age was per 10‐year increase.

In the sensitivity analysis including *BRCA1*/*2* data, similar trends were observed as 69.0% of the carriers reported ever having had an FBSE (Table S2). PV/LPV carriers had increased odds of FBSEs in the age‐adjusted model (AOR, 1.53; 95% CI, 1.05–2.22; *p* = 0.03); however, this association diminished in Model 2 after additionally controlling for demographic factors. Age and female sex were associated with increased odds of FBSEs; Black race, Hispanic ethnicity, and high school or less education were associated with lower odds of FBSEs (Table S3).

### 3.3 Family Letter Receipt, Familial Disclosure, and Cascade Testing

After genetic testing, 65.3% of carriers of PV/LPV in skin CPGs received a family letter from a genetic counselor or other clinicians, and 90.3% shared the letter with at least one family member (Table 3), with 55.2% and 35.8% having disclosed their PV/LPV‐carrying status to spouses or first‐degree relatives and second‐degree or extended family members, respectively. Further, 62.7% of the carriers reported that at least one family member underwent genetic testing, with 64.3% of the tests performed through clinical visits with a doctor or genetic counselor (Table 3). Metrics of family letter receipt, familial disclosure, and cascade testing in these high‐risk individuals were similar with the inclusion of *BRCA1*/*2* data (Table S2). Similar distributions of FBSE (82.0% vs. 72.0%; *p* = 0.71), family letter receipt (60.0% vs. 67%; *p* = 0.72), and cascade testing (91.0% vs. 71.0%; *p* = 0.26) by level of gene penetrance were also observed (Table S4).

**TABLE 3:** Percentages of family letter receipt, familial disclosure, and cascade testing in high‐risk individuals.

Characteristics	Overall	Carrier status of PV/LPV in skin cancer predisposition genes		*p* ^a^
		Non‐carriers	Carriers	
	No. (%)	No. (%)	No. (%)	
Most recent full‐body skin examinations				
Within the past year	200 (58.5)	171 (58.0)	29 (61.7)	0.97
Within the past 2 years (1 year but < 2 years)	61 (17.8)	53 (18.0)	8 (17.0)	
Within the past 3 years (2 years but < 3 years)	3 (6.4)	17 (5.8)	3 (6.4)	
Within the past 5 years (3 years but < 5 years)	28 (8.2)	24 (8.1)	4 (8.5)	
≥ 5 more years ago	32 (9.4)	29 (9.8)	3 (6.4)	
Don't know or don't remember	1 (0.3)	1 (0.3)	0	
Received a family letter from your genetic counselor or other clinicians after your genetic testing				
No	NA	NA	17 (34.7)	NA
Yes	NA	NA	32 (65.3)	
Shared the family letter with any of your family members				
No	NA	NA	3 (9.7)	NA
Yes	NA	NA	28 (90.3)	
Told any family members about your status of carrying PV/LPV(s) in skin cancer predisposition gene(s)				
No	NA	NA	0	NA
Yes	NA	NA	61 (91.0)	
Declined to answer/missing	NA	NA	6 (9.0)	
Relative whom you disclosed PV/LPV‐carrying status to				
Spouse and first‐degree	NA	NA	37 (55.2)	NA
Second‐degree and extended family	NA	NA	24 (35.8)	
Declined to answer/missing	NA	NA	6 (9.0)	
Any family members had genetic testing for the gene variant(s) identified in you				
No	NA	NA	14 (20.9)	NA
Yes	NA	NA	42 (62.7)	
Declined to answer/missing	NA	NA	11 (16.4)	
How did the family member receive the genetic testing?				
Clinical visits with a doctor or genetic counselor	NA	NA	27 (64.3)	NA
Used a consumer service (e.g., Ancestry, 23 and me, etc.)	NA	NA	4 (9.5)	
Received the test directly from a laboratory	NA	NA	7 (16.7)	
Don't know	NA	NA	4 (9.5)	

Abbreviations: NA, not applicable; No., number; PV/LPV, pathogenic/likely pathogenic variants.

^a^ *p* values were calculated using Pearson's chi‐squared or Fisher's exact tests.

## 4 Discussion

This cross‐sectional study of diverse high‐risk individuals demonstrates the potential role of PV/LPV in skin CPGs in relation to FBSEs. PV/LPV carriers had more than twice the odds of undergoing an FBSE than non‐carriers, possibly reflecting their increased awareness of cancer risk. This can be explained using the Health Belief Model (HBM) [44], an established and widely adopted theoretical framework that explains and predicts cancer risk prevention and behavioral change [45, 46, 47]. High‐risk individuals who receive genetic testing likely have higher perceptions of skin cancer risk in addition to their personal and/or family history. Knowing their status of PV/LPV in skin CPGs allows individuals to learn more about and gain control of their own risk. They perceive greater benefits of screening and are therefore more likely to undergo an FBSE. The intersections of PV/LPV in skin CPGs, perceived risk and benefits, and screening can be explored in future studies.

Additionally, significant disparities in the utilization of FBSEs were observed based on age, race/ethnicity, and educational attainment. Asian, Black, or Hispanic individuals and those with high school or less education exhibited notably lower odds of having an FBSE. While this may partially reflect group‐level differences in average risk, it also highlights a concerning trend: even among high‐risk individuals, some may not be accessing appropriate screening due to systemic or informational barriers. These results are associative and underscore the necessity of reducing disparities in skin cancer screening by ensuring access for all who may benefit and considering genetic testing for PV/LPVs in skin CPGs in high‐risk populations.

While previous research has primarily focused on environmental risk factors and sporadic melanoma cases [48], our study bridges a critical gap by investigating the utilization of FBSEs among high‐risk individuals, particularly carriers of PV/LPV in skin CPGs. We found > 70% of the carriers having received an FBSE and high rates of family letter receipt, familial disclosure of PV/LPV‐carrying status, and cascade genetic testing. However, despite this high uptake, only 54.8% of the carriers reported being informed by their providers that they were at risk for skin cancer. One in five carriers reported that none of their family members underwent genetic testing after being informed of the PV/LPV identified in skin cancer CPGs. These gaps underscore the need for improved patient‐provider communication regarding genetic risk and prevention. Psychological factors, cultural background, and health‐seeking behaviors may also contribute in part to the disclosure and testing gaps despite being aware of PV/LPV‐carrying status. Proper risk communication and psychosocial support are essential for informed decision‐making and risk assessment and management in the clinical setting.

Our findings also suggest that FBSEs could serve as a valuable tool for early detection, risk reduction, and management in high‐risk populations while considering PV/LPV carrier status beyond known risk factors when undergoing skin cancer screening. However, the evidence supporting skin self‐examination and the effectiveness of annual FBSEs in high‐risk populations remains limited [49]. The current AAD guidelines recommend that people who are at high risk should perform regular skin self‐exam (an A rank for strength of recommendation with a I/II level of evidence) and receive an annual skin check for melanoma, SCC, and BCC by a dermatologist [5, 6, 7]. Research is urgently needed to establish their clinical utility of testing for PV/LPV in skin CPGs and evaluate whether carrier status and screening have an impact on the health outcomes of high‐risk populations.

To build upon these findings, efforts should address disparities in FBSEs and how screening guidelines can be improved. Public health campaigns, clinical counseling, and genetic testing are critical components of a comprehensive prevention strategy. Understanding the interplay between risk factors, genetic counseling, and behaviors toward FBSEs is an essential area for future research. Studies have shown that genetic counseling for individuals with *CDKN2A* pathogenic variants can significantly influence their adherence to skin cancer prevention, including routine skin examinations and UV protection measures [50]. In addition to PV/LPVs in the *CDKN2A* gene, it is worth exploring how other high and low‐to‐moderate penetrance genes (i.e., *TP53*, *PTEN*, *BRIP1*, *PALB2*, *ATM*, and *CHEK2*) influence individuals' perceptions and screening behaviors. Maintaining long‐term behavioral change is challenging, as prior research indicates the limited sustainability of educational and genetic counseling interventions [51]. Investigating whether PV/LPV carriers who undergo genetic testing and counseling are more likely to adopt protective behaviors or increase FBSEs may help refine prevention strategies, given that > 60% of PV/LPV carriers' family members underwent genetic testing. Identifying these mechanisms could provide additional insights for integrating genetic and behavioral interventions to improve personalized screening approaches.

Increasing the awareness and knowledge of skin cancer screening, including FBSEs, remains important for improving preventive oncology care and services. In this study, we found that individuals who identified as Asian, Black, or Hispanic had significantly lower odds of having an FBSE than their White peers. These racial/ethnic differences may be largely driven by a higher incidence of skin cancer among White individuals in the US. This also may reflect the persistent inequitable care access and unequal health outcomes in racial/ethnic minority populations. A recent systematic review has demonstrated that Black or Hispanic patients often experience dermatologic care barriers, later‐stage diagnoses of skin cancer, and worse outcomes than their White peers [52]. Compared to individuals with a graduate or professional degree, those with a high school education or less had a lower likelihood of having had an FBSE. Our findings are consistent with previous research demonstrating disparities in access to and utilization of skin cancer screening in racially minoritized and/or socioeconomically disadvantaged populations [53, 54]. Taken together, this study suggests the need for education interventions and promotion for skin cancer screening. Further expanding affordable genetic testing tools and insurance coverage on comprehensive screening including FBSEs can be beneficial for disadvantaged or underserved populations. Efforts should prioritize these populations to ensure that preventive dermatologic care and services, regardless of PV/LPV carrier status.

### 4.1 Limitations

This study has several limitations. First, data were collected based on individual self‐report, which is subject to recall or social desirability bias. However, we would expect this bias to be very minimal because our research staff had little to no direct interaction with the participants at the time of the survey. This was unlikely to have influenced their survey responses. Self‐reported PV/LPVs were verified and confirmed by our clinical investigators to ensure accuracy. Second, there remain other unmeasured confounding factors, such as perceived risk of skin cancer, personal and/or family history of skin cancer, environmental exposures, occupation, provider recommendation, dermatological care access, and health‐seeking behaviors, that possibly better explain the differences observed in this study. Future research is worth investigating the impact of these and other potential factors on FBSEs among high‐risk individuals, particularly among PV/LPV carriers. In addition, this observational study used a cross‐sectional design, precluding us from determining a causal relationship between carrier status of PV/LPV in skin CPGs and FBSE utilization. Given the cross‐sectional study design, we could not definitively establish the temporality between PV/LPV testing and FBSE utilization. Lastly, our study sample may not be representative of all individuals at high risk for skin cancer and other high‐risk populations in the US. In particular, most of the CCPS participants were female, non‐Hispanic White, highly educated, and privately insured. Although racial/ethnic and socioeconomic disparities in FBSE utilization were observed, the generalizability of our findings is limited. But we would expect similar patterns of skin cancer screening uptake in diverse high‐risk populations who seek care at large urban medical centers. This study should be considered hypothesis‐generating. Larger prospective studies are necessary to further examine and confirm the benefits of population‐based genetic testing for skin cancer and how PV/LPV carrier status and social determinants inform skin cancer screening in US diverse populations.

## 5 Conclusions

In this multiethnic cohort of high‐risk individuals, carriers of PV/LPV in skin CPGs were more likely to undergo an FBSE than non‐carriers. Family letter receipt, familial disclosure, and cascade genetic testing were prevalent in high‐risk individuals. There were also notable racial/ethnic and socioeconomic differences in FBSEs. Our study highlights the potential role of PV/LPV carrier status on FBSEs in high‐risk populations. Future research should consider incorporating PV/LPV‐carrying status beyond known risk factors and how to optimize skin cancer screening practices and improve comprehensive guidelines.

## Funding

This work was supported in part by the Breast Cancer Research Foundation (BCRF‐23‐071), the US Department of Defense (W81XWH2210791), Susan G. Komen (TREND21675016 and SAC210203), the National Cancer Institute (P20CA233307 and R01CA228198), the National Institute on Aging (T32AG000243), KOSÉ Cosmetology Research Foundation Grant for International Exchange, and the 2023 Japan Cancer Society Relay for Life Award. The contents are solely the responsibility of the authors and do not necessarily represent the official views of the National Cancer Institute and the National Institute on Aging. The funding organizations had no role in the design and conduct of the study; collection, management, analysis, and interpretation of the data; preparation, review, or approval of the manuscript; and decision to submit the manuscript for publication.

## Ethics Statement

The University of Chicago Institutional Review Board reviewed and approved the current study.

## Consent

All study participants provided written informed consent to the Chicago Cancer Prone Study and follow‐up surveys.

## Conflicts of Interest

Dr. Olopade has declared financial relationships with CancerIQ and TempusAI; research funding from Color Genomics and Roche/Genentech. The other authors declare no conflicts of interest.

## Supporting information


**Figure S1:** CONSORT Diagram for Sample Selection of High‐Risk Individuals from the University of Chicago Cancer Prone Study (CCPS).
**Figure S2:** Number of Carriers of Pathogenic/Likely Pathogenic Variants (PV/LPV) in Skin Cancer Predisposition Genes.
**Table S1:** Sociodemographic Characteristics of High‐Risk Individuals, Overall and by Carrier Status of Pathogenic/Likely Pathogenic Variants in Skin Cancer Predisposition Genes (including *BRCA1* and *BRCA2*).
**Table S2:** Prevalence of Full‐Body Skin Examination, Familial Disclosure, and Cascade Testing, by Carrier Status of Pathogenic/Likely Pathogenic Variants in Skin Cancer Predisposition Genes (including *BRCA1* and *BRCA2*).
**Table S3:** Association between Carrier Status of Pathogenic/Likely Pathogenic Variants in Skin Cancer Predisposition Genes (including *BRCA1* and *BRCA2*) and Full‐Body Skin Examination among High‐Risk Individuals.
**Table S4:** Distributions of Full‐Body Skin Examination, Familial Disclosure, and Cascade Testing by Level of Skin Cancer Predisposition Gene Penetrance.

## Data Availability

The data that support the findings of this study cannot be shared publicly due to patient confidentiality and privacy concerns. However, the data can be de‐identified and made available upon reasonable request, pending from the approval of the University of Chicago Institutional Review Board and the corresponding authors.
