# Supplementary material for: Skin Cancer Predisposition Genes, Full‐Body Skin Examinations, Familial Disclosure, and Genetic Testing Among High‐Risk Individuals
Source: Int J Dermatol. 2025 Dec 10;65(7):1431–41. doi: 10.1111/ijd.70206 (PMC12818384; doi:10.1111/ijd.70206)
Supplement: Supplementary file 1 — Figure S1: CONSORT Diagram for Sample Selection of High‐Risk Individuals from the University of Chicago Cancer Prone Study (CCPS). Figure S2: Number of Carriers of Pathogenic/Likely Pathogenic Variants (PV/LPV) in Skin Cancer Predisposition Genes. Table S1: Sociodemographic Characteristics of High‐Risk Individuals, Overall and by Carrier Status of Pathogenic/Likely Pathogenic Variants in Skin Cancer Predisposition Genes (including BRCA1 and BRCA2). Table S2: Prevalence of Full‐Body Skin Examination, Familial Disclosure, and Cascade Testing, by Carrier Status of Pathogenic/Likely Pathogenic Variants in Skin Cancer Predisposition Genes (including BRCA1 and BRCA2). Table S3: Association between Carrier Status of Pathogenic/Likely Pathogenic Variants in Skin Cancer Predisposition Genes (including BRCA1 and BRCA2) and Full‐Body Skin Examination among High‐Risk Individuals. Table S4: Distributions of Full‐Body Skin Examination, Familial Disclosure, and Cascade Testing by Level of Skin Cancer Predisposition Gene Penetrance. [file IJD-65-1431-s001.pdf]

Supplementary Materials

Figure S1. CONSORT Diagram for Sample Selection of High-Risk Individuals from the University of Chicago Cancer Prone Study (CCPS)

Figure S2. Number of Carriers of Pathogenic/Likely Pathogenic Variants (PV/LPV) in Skin Cancer Predisposition Genes

Table S1. Sociodemographic Characteristics of High-Risk Individuals, Overall and by Carrier Status of Pathogenic/Likely Pathogenic Variants in Skin Cancer Predisposition Genes (including *BRCA1* and *BRCA2*)

Table S2. Prevalence of Full-Body Skin Examination, Familial Disclosure, and Cascade Testing, by Carrier Status of Pathogenic/Likely Pathogenic Variants in Skin Cancer Predisposition Genes (including *BRCA1* and *BRCA2*)

Table S3. Association between Carrier Status of Pathogenic/Likely Pathogenic Variants in Skin Cancer Predisposition Genes (including *BRCA1* and *BRCA2*) and Full-Body Skin Examination among High-Risk Individuals

Table S4. Distributions of Full-Body Skin Examination, Familial Disclosure, and Cascade Testing by Level of Skin Cancer Predisposition Gene Penetrance

Figure S1. CONSORT Diagram for Sample Selection of High-Risk Individuals from the University of Chicago Cancer Prone Study (CCPS)

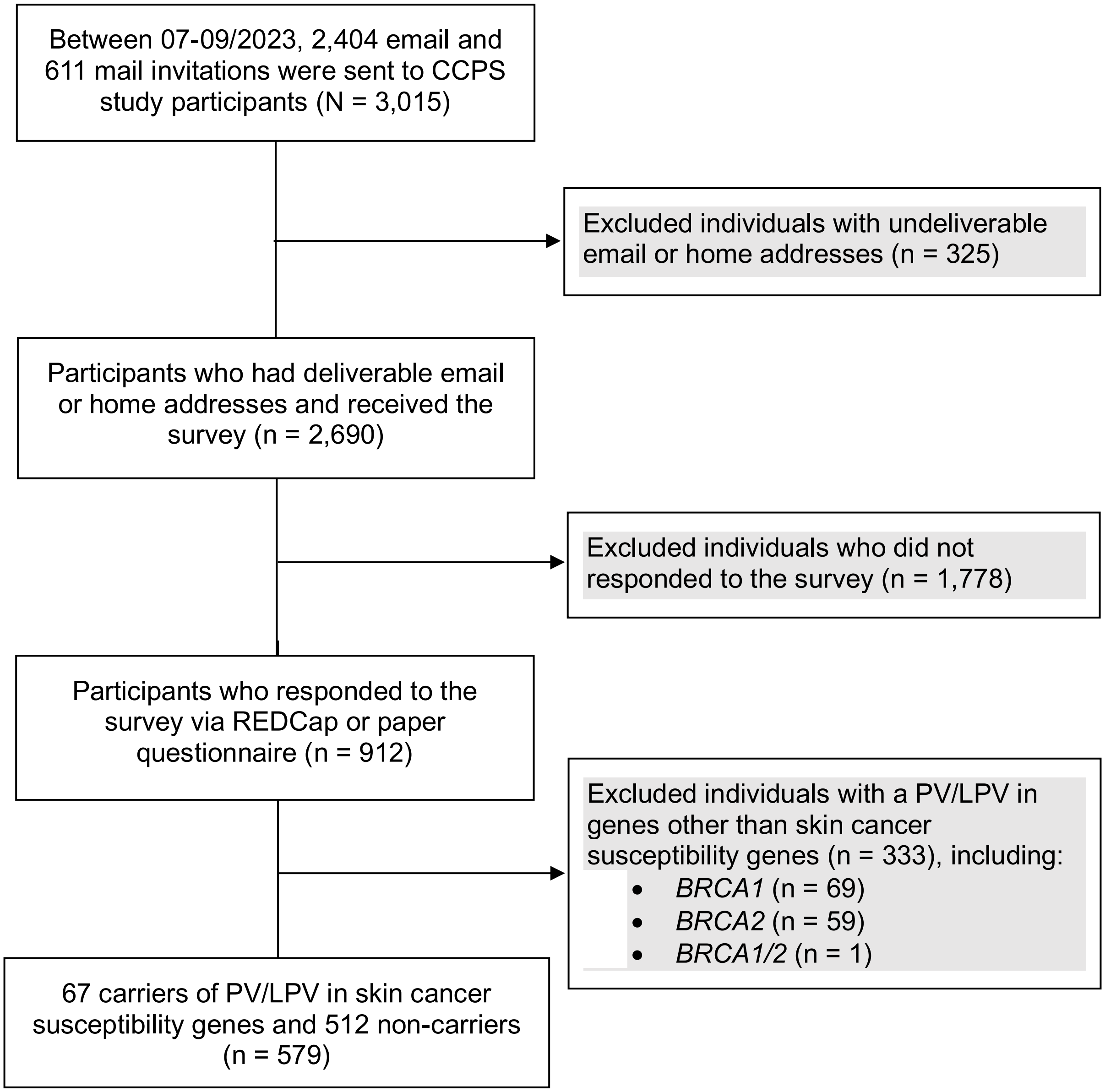

Figure S2. Number of Carriers of Pathogenic/Likely Pathogenic Variants (PV/LPV) in Skin Cancer Predisposition Genes

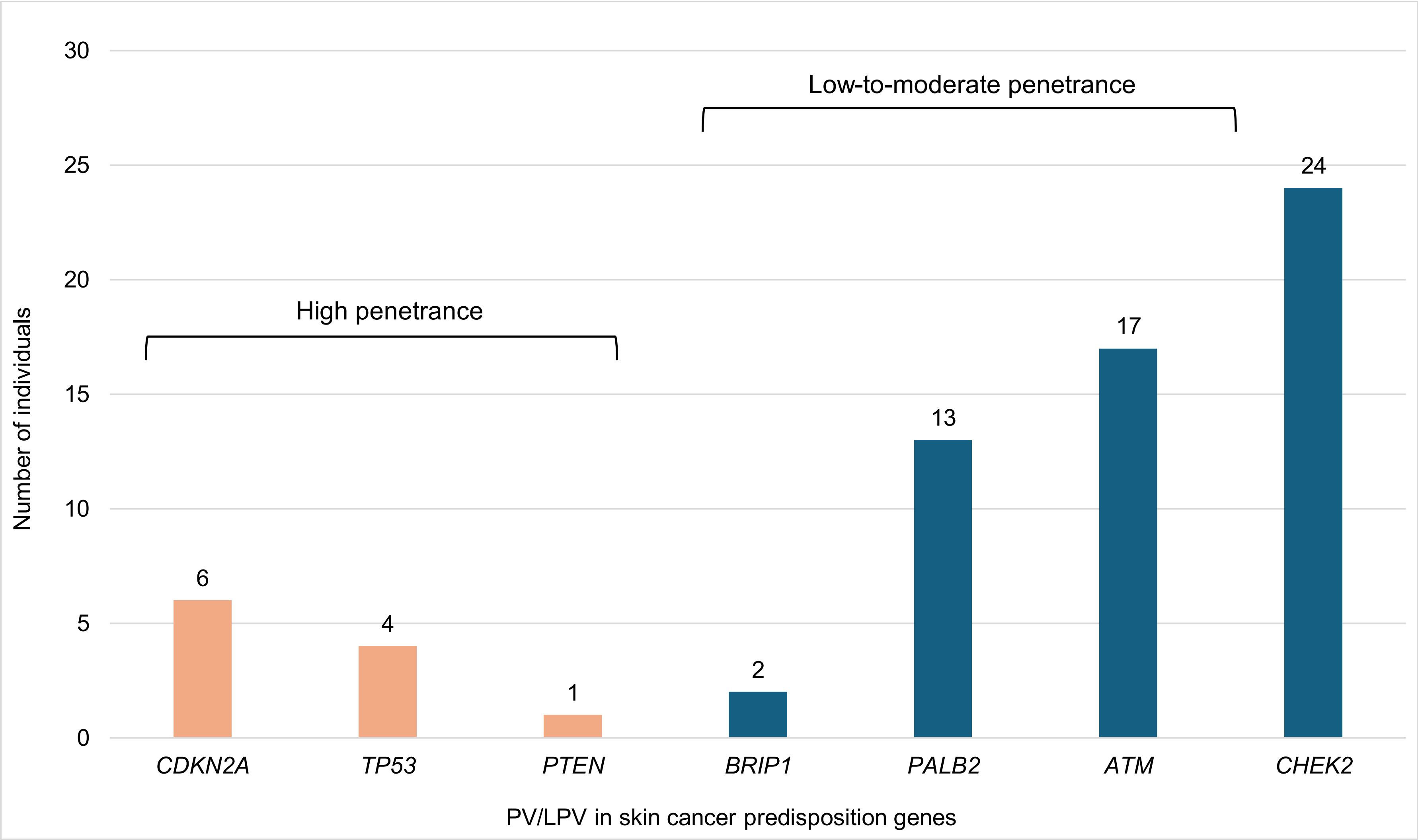

Table S1. Sociodemographic Characteristics of High-Risk Individuals, Overall and by Carrier Status of Pathogenic/Likely Pathogenic Variants in Skin Cancer Predisposition Genes (including *BRCA1* and *BRCA2*)

		**Carrier status of PV/LPV in skin cancer predisposition genes** (including *BRCA1* and *BRCA2*)		
	**Overall**	**Non-Carriers**	**Carriers**	
**Characteristic**	**N = 706**	**n = 512 (72.5%)**	**n = 194 (27.5%)**	
	**No. (%)**	**No. (%)**	**No. (%)**	***P* Value [a]**
**Age in years**				
Mean (SD)	58.9 (13.3)	60.0 (13.0)	55.8 (13.6)	<.001
Median (IQR)	60.0 (50.0, 69.0)	61.0 (51.0, 70.0)	57.0 (45.0, 65.0)	<.001
**Sex assigned at birth**				
Male	125 (17.7)	92 (18.0)	33 (17.0)	.77
Female	581 (82.3)	420 (82.0)	161 (83.0)	
**Race/ethnicity**				
Non-Hispanic Asian	23 (3.3)	15 (3.0)	8 (4.2)	.16
Non-Hispanic Black	70 (10.2)	58 (11.7)	12 (6.3)	
Non-Hispanic White	568 (82.7)	404 (81.5)	164 (85.9)	
Hispanic	26 (3.8)	19 (3.8)	7 (3.7)	
**Marital status**				
Married or living with a partner	493 (71.9)	352 (71.1)	141 (73.8)	.42
Single or never married	72 (10.5)	50 (10.1)	22 (11.5)	
Widowed, divorced or separated	121 (17.6)	93 (18.8)	28 (14.7)	
**Highest level of education**				
High school, GED or less	51 (7.4)	35 (7.0)	16 (8.4)	.31
Some college or Associate's degree	111 (16.1)	80 (16.1)	31 (16.2)	
Bachelor's degree	204 (29.7)	157 (31.6)	47 (24.6)	
Graduate or professional degree	322 (46.8)	225 (45.3)	97 (50.8)	
**Type of health insurance**				
Private	429 (62.4)	299 (60.3)	130 (68.1)	.06
Medicaid	7 (1.0)	3 (0.6)	4 (2.1)	
Medicare	216 (31.4)	169 (34.1)	47 (24.6)	
Other or other government plan	28 (4.1)	20 (4.0)	8 (4.2)	
Uninsured	7 (1.0)	5 (1.0)	2 (1.0)	
**Annual household income**				
<$50,000	66 (10.2)	45 (9.7)	21 (11.6)	.46
$50,000 – $74,999	72 (11.2)	51 (11.0)	21 (11.6)	
$75,000 – $99,999	82 (12.7)	55 (11.9)	27 (14.9)	
$100,000 – $149,999	132 (20.5)	103 (22.2)	29 (16.0)	
$150,000 – $199,999	93 (14.4)	64 (13.8)	29 (16.0)	
≥$200,000	200 (31.0)	146 (31.5)	54 (29.8)	
**Smoking status**				
Current	17 (2.5)	13 (2.7)	4 (2.1)	.86
Past	180 (26.9)	131 (27.4)	49 (25.8)	
Never	471 (70.5)	334 (69.9)	137 (72.1)	
**Ever told by a doctor or other health care provider that you are at risk for skin cancer**				

No	425 (65.3)	333 (70.6)	92 (51.4)	<.001
Yes	226 (34.7)	139 (29.4)	87 (48.6)	

Abbreviations: PV/LPV, pathogenic/likely pathogenic variants; No., number; SD, standard deviation: IQR, interquartile range; GED, general education development.
[a] *P* values were calculated using Student's t, Wilcoxon rank-sum, Pearson's Chi-squared, or Fisher's exact tests, as appropriate.

Table S2. Prevalence of Full-Body Skin Examination, Familial Disclosure, and Cascade Testing, by Carrier Status of Pathogenic/Likely Pathogenic Variants in Skin Cancer Predisposition Genes (including *BRCA1* and *BRCA2*)

		**Carrier status of PV/LPV in skin cancer predisposition genes** (including *BRCA1* and *BRCA2*)		
**Characteristic**	**Overall**	**Non-Carriers**	**Carriers**	
	**No. (%)**	**No. (%)**	**No. (%)**	***P* Value [a]**
**Ever had a full-body skin examination**				
No	235 (35.7)	177 (37.5)	58 (31.0)	.12
Yes	129 (64.3)	295 (62.5)	129 (69.0)	
**Most recent full-body skin examination**				
Within the past year	249 (58.9)	171 (58.0)	78 (60.9)	1.0
Within the past 2 years (1 year but <2 years)	74 (17.5)	53 (18.0)	21 (16.4)	
Within the past 3 years (2 years but <3 years)	24 (5.7)	17 (5.8)	7 (5.5)	
Within the past 5 years (3 years but <5 years)	34 (8.0)	24 (8.1)	10 (7.8)	
≥5 more years ago	41 (9.7)	29 (9.8)	12 (9.4)	
Don't know or don't remember	1 (0.2)	1 (0.3)	0	
**Received a family letter from your genetic counselor or other clinicians after your genetic testing**				
No	NA	NA	47 (35.9)	NA
Yes	NA	NA	84 (64.1)	
**Shared the family letter with any of your family members**				
No	NA	NA	10 (13.2)	NA
Yes	NA	NA	66 (86.8)	
**Told any family members about your status of carrying PV/LPV(s) in skin cancer predisposition gene(s)**				
No	NA	NA	0	NA
Yes	NA	NA	180 (100)	
**Relative whom you disclosed PV/LPV-carrying status to**				
Spouse and first-degree	NA	NA	81 (45.5)	NA
Second-degree and extended family	NA	NA	97 (54.5)	
**Any family members had genetic testing for the gene variant(s) identified in you**				
No	NA	NA	23 (13.3)	NA
Yes	NA	NA	150 (86.7)	
**How did the family member receive the genetic testing?**				
Clinical visits with a doctor or genetic counselor	NA	NA	108 (75.5)	NA
Used a consumer service (e.g., Ancestry, 23andme, etc.)	NA	NA	7 (4.9)	
Received the test directly from a laboratory	NA	NA	12 (8.4)	
Don't know	NA	NA	16 (11.2)	

Abbreviations: PV/LPV, pathogenic/likely pathogenic variants; No., number; NA, not applicable.
[a] *P* values were calculated using Pearson's Chi-squared or Fisher's exact tests.

Table S3. Association between Carrier Status of Pathogenic/Likely Pathogenic Variants in Skin Cancer Predisposition Genes (including *BRCA1* and *BRCA2*) and Full-Body Skin Examination among High-Risk Individuals

	**Multivariable logistic regression**			
**Characteristic**	**Model 1**		**Model 2**	
	**AOR (95% CI)** [a]	***P* Value**	**AOR (95% CI)** [b]	***P* Value**
**Carrier status of PV/LPV in skin cancer predisposition genes** (including *BRCA1* and *BRCA2*)				
Carriers	1.53 (1.05-2.22)	.03	1.26 (0.81-1.95)	.31
Non-Carriers	1 [reference]		1 [reference]	
**Age in years** [d]	1.31 (1.16-1.49)	<.001	1.38 (1.12-1.69)	.002
**Sex assigned at birth**				
Male	NA	NA	1 [reference]	
Female	NA	NA	2.03 (1.22-3.37)	.006
**Marital status**				
Married or living with a partner	NA	NA	1 [reference]	
Single or never married	NA	NA	0.52 (0.26-1.06)	.07
Widowed, divorced or separated	NA	NA	0.73 (0.40-1.32)	.30
**Race/ethnicity**				
Non-Hispanic Asian	NA	NA	0.45 (0.17-1.15)	.10
Non-Hispanic Black	NA	NA	0.04 (0.02-0.10)	<.001
Non-Hispanic White	NA	NA	1 [reference]	
Hispanic	NA	NA	0.16 (0.06-0.41)	<.001
**Highest level of education**				
High school, GED or less	NA	NA	0.28 (0.13-0.64)	.002
Some college or Associate's degree	NA	NA	0.61 (0.34-1.09)	.10
Bachelor's degree	NA	NA	0.79 (0.50-1.27)	.34
Graduate or professional degree	NA	NA	1 [reference]	
**Annual household income**				
<$50,000	NA	NA	0.54 (0.22-1.32)	.18
$50,000 – $74,999	NA	NA	0.44 (0.21-0.95)	.04
$75,000 – $99,999	NA	NA	0.88 (0.42-1.83)	.74
$100,000 – $149,999	NA	NA	0.66 (0.37-1.19)	.17
$150,000 – $199,999	NA	NA	0.58 (0.31-1.06)	.08
≥$200,000	NA	NA	1 [reference]	
**Type of health insurance**				
Private	NA	NA	1 [reference]	
Medicaid	NA	NA	1.59 (0.15-17.11)	.70
Medicare	NA	NA	1.21 (0.65-2.26)	.55
Other or other government plan	NA	NA	1.12 (0.40-3.13)	.84

Abbreviations: PV/LPV, pathogenic/likely pathogenic variants; AOR, adjusted odds ratio; CI, confidence interval; GED, general education development; NA, not applicable.

[a] adjusted for age only.

[b] adjusted for age, sex assigned at birth, race/ethnicity, marital status, highest level of education, annual household income, and type of health insurance coverage.

[c] The AOR and 95% CI for age was per 10-year increase.

Table S4. Distributions of Full-Body Skin Examination, Familial Disclosure, and Cascade Testing by Level of Skin Cancer Predisposition Gene Penetrance

	**High penetrance**	**Low-to-moderate penetrance**	
**Characteristic**	PV/LPVs in *CDKN2A*, *TP53*, and *PTEN* genes (n = 11)	PV/LPVs in *BRIP1*, *PALB2*, *ATM*, and *CHEK2* (n = 56)	
	**No. (%)**	**No. (%)**	***P* Value [a]**
**Ever had a full-body skin examination**			
No	2 (18)	15 (28)	.71
Yes	9 (82)	38 (72)	
**Most recent full-body skin examination**			
Within the past year	8 (89)	21 (55)	.65
Within the past 2 years (1 year but <2 years)	1 (11)	7 (18)	
Within the past 3 years (2 years but <3 years)	0	3 (8)	
Within the past 5 years (3 years but <5 years)	0	4 (11)	
≥5 more years ago	0	3 (8)	
**Received a family letter from your genetic counselor or other clinicians after your genetic testing**			
No	4 (40)	13 (33)	.72
Yes	6 (60)	26 (67)	
**Shared the family letter with any of your family members**			
No	1 (17)	2 (8)	.49
Yes	5 (83)	23 (92)	
**Told any family members about your status of carrying PV/LPV(s) in skin cancer predisposition gene(s)**			
Yes	11 (100)	50 (100)	NA
**Relative whom you disclosed PV/LPV-carrying status to**			
Spouse and first-degree	5 (45)	32 (64)	.32
Second-degree and extended family	6 (55)	18 (36)	
**Any family members had genetic testing for the gene variant(s) identified in you**			
No	1 (9)	13 (29)	.26
Yes	10 (91)	32 (71)	
**How did the family member receive the genetic testing?**			
Clinical visits with a doctor or genetic counselor	7 (70)	20 (62)	.44
Used a consumer service (e.g., Ancestry, 23andme, etc.)	2 (20)	2 (6)	
Received the test directly from a laboratory	1 (10)	6 (19)	
Don’t know	0	4 (12)	

Abbreviations: PV/LPV, pathogenic/likely pathogenic variants; No., number; NA, not applicable.

[a] *P* values were calculated using the Fisher’s exact test.
